# Identification of causes and consequences of Kolberi among Iranian Kurdish women: a grounded theory study

**DOI:** 10.1186/s12905-023-02791-5

**Published:** 2023-12-05

**Authors:** Javad Yoosefi Lebni, Seyed Fahim Irandoost, Arash Ziapour, Ahmad Ahmadi, Nafiul Mehedi, Seyyed Amar Azizi

**Affiliations:** 1 Social Determinants of Health Research Center, Lorestan University of Medical Sciences, Khorramabad, Iran; 2 Department of Community Medicine, School of Medicine, Urmia University of Medical Sciences, Urmia, Iran; 3 Cardiovascular Research Center, Health Institute, Kermanshah University of Medical Sciences, Kermanshah, Iran; 4 Faculty of Psychology and Educational Sciences, Allameh Tabataba’i University, Tehran, Iran; 5 Department of Social Work, Shahjalal University of Science and Technology, Sylhet, Bangladesh; 6 Students Research Committee, Kermanshah University of Medical Sciences, Kermanshah, Iran; 7 University of Social Welfare and Rehabilitation Sciences, Tehran, Iran

**Keywords:** Kolberi, Women, Causes, Consequences, Kurdistan, Grounded theory

## Abstract

**Background:**

Women turn to Kolberi for various reasons, which cause numerous challenges for them. Thus, it is imperative to identify these causes and problems. Since no study has ever been undertaken to deal with this participant, the present research aims to identify the causes and consequences of Kolberi among Kurdish women in Iran.

**Method:**

The present research uses the grounded theory approach to investigate 28 female Kurdish Kolbers. To achieve several participants, purposive, snowball, and theoretical sampling methods were used, while face-to-face semi-structured interviews were used to gather data. The process of data collection and analysis took 10 months, from April to December 2022. The data were analyzed using the Strauss and Corbin method and MAXQDA-20018 software. The Guba and Lincoln criteria were also met to increase the trustworthiness of the results.

**Findings:**

Analysis of the data led to 143 initial codes, 31 subcategories, and 9 main categories: Causal condition (individual characteristics and economic factors); predisposing conditions (social and cultural factors, familial factors); intervening conditions (advantages and characteristics of Kolberi); strategies (strengthening compatibility with Kolberi); and consequences (individual problems, social problems and positive consequences).

**Conclusion:**

Measures such as training occupational skills for women and providing employment conditions for them, increasing social, financial, and mental support for women without guardians, creating border markets, and expanding women's handicrafts can help prevent female Kolberi.

## Introduction

The western provinces of Iran (West Azerbaijan, Kurdistan, Kermanshah, and Ilam), which share borders with Iraq, have always been places of conflict and bloody wars, causing destruction and disruption of existing infrastructure. Also, damages caused by the Iraq-Iran war and the U.S.-led invasion of Iraq back in 2003 led to the insecurity of border areas in these provinces, resulting in the loss of local investment and non-investment at local, national, and international levels. These negative consequences have, since then, caused underdevelopment in these regions [1, 2]. This situation culminated in unemployment in these regions, which was followed by social and economic crises, which also caused migration to other developed areas of the country, rising divorce cases, poverty, the class gap, smuggling, and false employment (Kolberi) [3, 4].

One of the ways that people in these regions earn a living is through Kolberi, the local language. Piggybacking refers to the act of carrying foreign-made goods on one’s back over the borders without a customs license to earn a living against an insignificant amount of money [5–7]. Kolbers are from the western borders of Iran who transport goods through the borders on their backs without using any other means [8].

There is no written document over the history of this phenomenon in Kurdish regions; however, one would say that people of the border regions used to make money by performing barter exchanges and border trading in the past, as goods were used to be transported over the backs of animals before the formation of the modern state. Today, however, due to legal restrictions levied by states, border residents use their own forces to transport goods [9]. Statistics about the number of Kurdish Kolbers are estimated to range from 70.000 to 200.000 [4, 10], mostly working in Kurdistan, Western Azerbaijan, and Kermanshah [4].

The phenomenon of Kolberi, believed to be a job by locals [8], has an official and unofficial form; the official form refers to the carriage of goods of a certain amount at a certain interval at official border markets, which is performed by people with Kolberi cards based on approved laws. The unofficial form also refers to the carriage of goods by people through international borders in an illegal way [1]. Various factors, including unemployment, poverty, unsuitable environmental conditions, low education and lack of skills and expertise, lack of investment and exit of local investment from these regions, as well as the benefit of special groups from Kolberi at border markets, are involved in the creation and expansion of Kolberi [2, 4, 5, 7, 9, 11]. One of the important factors contributing to the emergence and continuation of the phenomenon of Kolberi in Iran is the border proximity with Iraqi Kurdistan, which facilitates Kolberi through shared linguistic, religious, ethnic, and cultural identities, as well as various forms of mobility, communication, and trade exchanges.

Anbari and Abedzadeh (2020) did a study on Kurdish regions in Iran, reporting that economic poverty and lack of alternative income sources, a sense of structural discrimination, environmental determinism, weak employment occupations, poor agriculture and tourism sectors, and the government’s failure to provide appropriate support for the poor of these regions are among the causes of Kolberi [9]. Also, domestic demands caused by shortage and undesirability of domestic products and high benefits of smuggled goods, government economic ineffectiveness, instability of neighboring states, residents of various groups at borders, religious and cultural similarities of the two bordering countries, long borders, distance of border regions from the central areas, and officials’ weak intelligence about the problems of these regions are some other reasons behind the phenomenon of Kolberi [8]. Torgler and Schneider (2009) also did a study and concluded that rising unemployment in the official sector increases the number of people who work in the unofficial sector and the black market [12]. Janparvar et al. (2021) also mentioned the policies of the two countries of Iran and Iraq, earning a livelihood, unemployment, and a lack of farmlands as the most important reasons for turning to Kolberi [2].

Kolberi is associated with numerous consequences due to its conditions; for example, as an unofficial and smuggling phenomenon, it reduces domestic occupational opportunities, reduces government revenues, causes stagnation and bankruptcy of the domestic industries, money laundering, policy failures, and ineffective government decisions [13]. Zareshahabadi and Mohammadi (2021) did a study in the city of Baneh (a Kurdish city in Kurdistan, Iran) to find that Kolbers were facing such problems as violence, occupational dissatisfaction, low morale, route insecurity, and job burnout [5]. In the meantime, Kolbers are forced to go through long mountainous routes (several kilometers) with heavy loads, thus risking such dangers as freezing, getting caught in an avalanche or blizzard, falling from heights, stepping on individual mines, etc. [1, 11]. It was found that from 2012 until December 3, 2021, 621 Kolbers were killed and 1088 were wounded [10, 14]. This figure is probably much higher, as many of the fatalities remain hidden due to security considerations and fear of being interrogated.

Despite the fact that Kolberi is, in principle, a male activity, women have, in the recent decade, also turned to this profession. Recently, women, mostly female heads of households, have joined male Kolbers. Some of them are widows, and others have the responsibility of taking care of their husbands, who have become disabled from carrying loads. They try to prove that they are capable of performing this arduous work by delivering the load safely to its destination. These women risk their lives every day to make a meagre living by carrying heavy loads across borders. They carry mobile phones, televisions, clothing, vacuum cleaners, tires, cigarettes, dried tea, satellite dishes, etc. They face harsh weather and mountainous paths and risk their lives to provide for their families and avoid starvation. Moreover, women cannot work during the winter since load owners believe they do not have the strength and physical power to survive in the severe weather and that their deaths will cause the owners' loads to be lost [14]. There is no accurate figure for female Kolbers, as they have been less focused due to some social and cultural considerations and fear of imprisonment by police forces.

Considering the cause and expanded consequences of Kolberi, it is important to conduct a comprehensive study on the causes, strategies, and consequences of Kolberi. Also, all the research conducted on Kolberi has addressed men, as no academic contributions have ever addressed female Kolbers, indicating the significance of doing this research. The reason for this significance comes from the fact that women's tendency to Kolberi may be different from that of men, as the experience and consequences of Kolberi for women are different from those of men. For this reason, the need to investigate the phenomenon of Kolberi from women's points of view is a focus of attention, and based on this, the present research was conducted with the aim of explaining the process of the emergence of the Kolberi phenomenon and consequences of Kolberi among Iranian Kurdish Women. The main question of the research was how the phenomenon of Kolberi forms among Kurdish women and how they interact with it.

## Methods

### Design

The present research was performed by using one of the qualitative research strategies, i.e., grounded theory, and relying on Strauss and Corbin’s systematic approach. Since the phenomenon of women Kolberi is a multi-dimensional and complex issue, it should be explained using a qualitative approach and a grounded theory method because the qualitative method is the best-case scenario for understanding complex phenomena [15]. Also, since grounded theory is a research tool that can explore and model hidden layers of social structures through constant comparison and is used for the gathering and analysis of data aimed at providing a theory, it is the best method to be used [16].

### Participants

The participants were females who were Kolberi in border regions of the western provinces of Kurdistan, Kermanshah, and parts of West Azarbaijan, Iran. Criteria of inclusion were being Kurd, having an experience of Kolberi in the recent month, Kolberi in border Kurdish regions, and a tendency to participate in the study; criteria of exclusion were a lack of a tendency to participate in the study and withdrawal from continuing with the interview.

### Data gathering

The sampling method began with the purposive technique, and snowball sampling was also used later [17]. Because the researchers were locals of the area under study, they identified seven female Kolbers and coordinated with them to conduct the interview process. Consistent with the snowball sampling, the participating women were required to explain to the researcher the criteria for inclusion in the study. Because Kolberi is considered a criminal act and many of the participants could not be identified due to security and cultural considerations, the snowball method was used to include 13 women. In the theoretical sampling method (selection of 8 people), following the identification of initial classes, selecting the next participant was based on the principle of how much she could help clarify the next classes [18]. Data were gathered through semi-structured interviews, which were conducted face-to-face without the intervention of another person except for the researcher. In order for the participants to share their experiences more easily with the researcher, a woman trained and skilled in qualitative interviewing with a master's degree in sociology was used to collect data. The time and place of the interviews were determined by the participants, as the interviews were conducted at the women’s personal houses in the evenings or afternoons. Each interview lasted on average 45 min, as the mean total time was 56 min. The process of data collection and analysis took 10 months, from April to December 2022. An interview item guide was used to gather data. This guide was developed in three sessions by the research team in three experimental interviews, which were finally completed with the modification of some sections (Table 1). There were some questions in the guide that were asked by the participants. All the questions of the interview were asked to the participants, but the order of the questions and the duration of the interview were different according to the answers given by the participants.

**Table 1:** Interview question guide with female participants of the research

No	Questions
1	What made you turn to Kolberi? Please explain
2	How much are you interested in this job? Did you choose this out of your interest or out of compulsion? Please explain
3	What are the advantages and disadvantages of Kolberi compared to other works?
4	How do you feel as a female Kolber?
5	How did your family and relatives react to you when you first piggybacked?
6	What is the reaction of the family, relatives and other Kolbers to you?
7	How do the owner of the load, border officials and another Kolberi treat with you? Please explain
8	How do you feel to see most of your colleagues are men?
9	What problems did you face when you turned to Kolberi?
10	What are the most important fears and concerns of you when Kolberi?
11	Has Kolberi disrupted your family relations and your roles in the family as a woman and a wife or a mother? Please explain
12	How do you cope with problems from Kolberi?

Interviews, data gathering, and analysis continued until theoretical saturation was achieved. Theoretical saturation is when no new codes are formed in the interviews and all the codes are repeated. In this research, theoretical saturation was achieved by interviewing 28 people.

### Data analysis

**Fig. 1:**
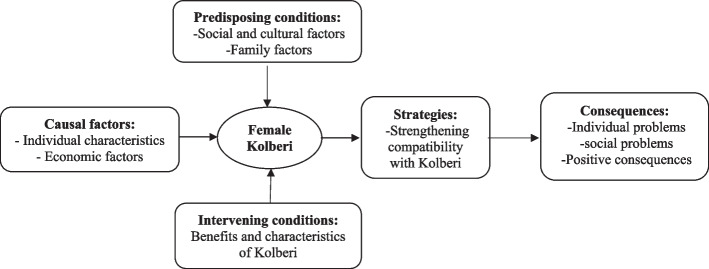
Conceptual model of the formation of the phenomenon of women's Kolberi (Unpleasant coexistence of poverty, environment, and patriarchy)

The MAXQDA-2018 software was used for data management. The data were analysed based on Strauss and Corbin’s approach. In this approach, data analysis is conducted through three stages of coding: open coding, axial coding, and selective coding. Open coding is a process in which data are categorized into distinct meaningful units that can be used at the beginning of the study. The main goal of open coding is conceptualization and labeling of the data. During the data analysis phase and in the search for codes, open coding becomes feasible. The second stage is axial coding, which involves the process of relating subcategories to more central categories. In this stage, among the codes obtained from open coding, those that appear to be more relevant than others in the subsequent stages are selected. Finally, selective coding is the process of selecting a core category and connecting all other categories to that core category. The main idea is to expand the central storyline around which everything takes shape [16]. After conducting each interview, the first and second authors translated it from Kurdish to Persian and typed it in word 2016 software. Simultaneously, the analysis process began, and the data acquired was used to prepare the next questions from the interviewees. This process continued until the end of the interviews. In the open coding stage, which is a kind of microscopic analysis of data, all possible meanings were broken down for consideration. In the second stage, which was the axial stage, initial codes and categories created in open coding were compared. In the meantime, when integrating similar cases, the categories that were related fell under a common axis, and the constant comparison of the codes was meticulously performed in this stage (Table 2). Then, two researchers compared each category with the other to ensure that the categories were different from each other. Then, by focusing on conditions that led to the phenomenon of female Kolberi, the grounds that were laid to create it and strategies used to control it were determined using selective coding. In fact, in the selective coding, categories were integrated and refined, and the main model of female Kolberi was formed (Fig. 1).

**Table 2:** An example of data analysis

Categories	Subcategories	Codes	Quotations
Economic factors	Financial poverty	Poverty, financial crunch, debts, low support from social organizations	“We have no land nor house. We have rented a house and cannot afford to meet our needs. We need to do Kolberi” “Life incurs much costs, and we have no income. My children are small and can’t work. I have to go Kolberi”
	Unemployment	Lack of jobs, widespread family unemployment, widespread unemployment in the region, lack of farmlands and fertile lands, lack of industrial workshops in the region, lack of investment for launching a job	“There is no other job to do; otherwise, I would never do Kolberi” “If there is someone to come forward and give me a good job, I will do away with Kolberi” “There are no other jobs in here, no factories, no workshop, nothing. Our farming also has little income and we have to go Kolberi” “I have no money to get a job around. I have to come and do the Kolberi”

### Trustworthiness

To ensure the Trustworthiness of the results, Guba and Lincoln's criteria were met [19]. For the data to be credible, the researcher’s lengthy involvement with the research scope had to be maintained. Since the researchers were from the area under study, two of whom had actually experienced Kolberi, this involvement was made with the research. At the end of each interview, the researcher expressed his general perception of the participants’ quotations to be confirmed by them. Also, research findings were provided to five participants to be confirmed. The researchers also tried to choose samples with highly diverse demographic characteristics. Conformability was met by observing the researcher’s impartiality and agreement with all the article authors and experts with knowledge of the qualitative research and the issue of Kolberi as regards the codes, categories, and subcategories. All raw data and notes, as well as the documents, were protected for later revisions. To achieve dependability in all stages of the research, the views of all authors who were from various scientific disciplines were used, and the titles of the categories and subcategories in the analysis process were finalized following consultation among all the authors. For transformability, a comprehensive description of the participants, including their demography, was offered first. Also, when analyzing the data, deep descriptions of the conditions were made. Many direct quotations were made by the participants for each of the categories and subcategories.

### Ethical considerations

Research ethical considerations include receiving a code of ethics and gaining permission to enter the research, the expression of study goals and its procedures for the Participants, ensuring the confidentiality of the data, like names, addresses of residency, etc., and gaining informed written consent forms to attend the research and record the dialogues, as well as the right to withdraw from the research at any time.

## Results

In this research, 28 Kolberi women from Kurdish regions of Iran participated. Most of the participants were illiterate, rural, widowed or divorced, and aged 20–40 years (Table 3). After data analysis, categories, subcategories, and paradigmatic structure were established (Fig. 1), and generally 143 primary codes, 31 subcategories, and 9 main categories were derived (Table 4).

**Table 3:** Demographic characteristics of Kolber women

Variable	Categorization	Frequency
Age	Under 20 years	4
	20–40 years	13
	Over 40 years	11
Marriage status	Married	8
	Single	6
	Divorced/widow	14
Residency	City	11
	Village	17
Education	Illiterate	12
	Under diploma	9
	Diploma	5
	Higher than diploma	2
History of Kolberi by year	Less than a year	4
	1–3 years	9
	3–6 years	10
	Over 6 years	5
Number of times of Kolberi per week	0–1	5
	2–4	16
	Over 4 times	7
Weight of the load carried (Kg)	Least	12
	Highest	40
	Mean	24

**Table 4:** Categories, subcategories and codes obtained from interviews with Kolber women

Paradigm structure	Categories	Subcategories	Codes
Causal conditions	Individual characteristics	Low empowerment	Illiteracy, lack of sufficiency expertise
		Personality characteristics	Tendency to high income at less time, being emotional, tendency to independence, risk-taking, hardworking
		Previous experience	Having a history of mountaineering, history of sheepherding in mountains, history of brining water from mountainous springs, history of carrying loads and displacing agricultural products and gathering edible plants in the mountains
		Considering Kolberi as ethical	Not recognizing Kolberi as a crime, considering Kolberi as lawful, considering Kolberi as useful for both countries
		Considering Kolberi as a sports or hobby	Enjoying beautiful routes of the mountain, especially in springs and autumns, avoiding the monotonous environment of the home, doing Kolberi as a hobby or sport, mobility
	Economic factors	Financial poverty	Poverty, financial crunch, debts, low support from social organizations
		Unemployment	Lack of jobs, widespread family unemployment, widespread unemployment in the region, lack of farmlands and fertile lands, lack of industrial workshops in the region, lack of investment for launching a job
Predisposing conditions	Social and cultural factors	Social learning	Learning Kolberi from friends, familiarity with Kolberi in the community, learning Kolberi from the family
		Social interactions and bonds	Having large-scale social relations with the country of Iraq, familial commuting with Iraqi Kurds, marriage relations with Iraqi Kurds, linguistic and cultural similarities, and social relations with Iranian and Iraqi Kurds
	Familial factors	Lack of supporter	No guardians, no husbands, being a widow, divorced, having a crippled husband
		Familial compulsion	High population in the family, having a retarded child in the family
Intervening conditions	Advantages and characteristics of Kolberi	Appropriate income	Immediate wages in cash for Kolberi, higher income of Kolberi compared to other occupations in the region
		Working independence	Setting time for Kolberi by the individual, determining the number of times of Kolberi by the individual
		Being addictive	Being accustomed to Kolberi, dependence on Kolberi, the tendency to continue Kolberi
		Receive support during Kolberi	Men’s assistance of female Kolber, being ignored by officials, officials’ milder treatment of the women, more attention and support by the load owner to women, attention and support of the buyers to purchasing loads from women than from men
Strategies	Strengthening compatibility with Kolberi	Taking a male identity	Taking male gestures, wearing male clothes, talking like men, smoking
		Concealment	Separation from the family for hiding the Kolberi work, hiding Kolberi from the family, attempts to hide Kolberi from relatives, masking one’s face when doing Kolberi, use of intermediaries for taking delivery of loads
		Formation of female groups	Accompanying female counterparts in Iraq, trading with women in Iran and Iraq,
		More communication with God	Praying with God, trust in God, almsgiving in the path of God
Consequences	Individual problems	Physical harms	Backache, headache, knee ache, neck ache, shoulder dislocation, amputation
		Mental issues	Anxiety, sever stress, humiliation, loss of self-confidence, Permanent fear and distress of having the loads confiscated by the officials, fear of losing face, fear of having one’s job revealed to the family and others, fear of receiving harm from the animals
		Loss of life security	Risk of falling from the cliffs, being caught by typhoons and avalanches, risk of being killed or wounded by the officials, freezing, crashing when moving the loads
		Addiction	Turning to drugs for gaining more energy, addiction to energetic drugs, addiction to drugs, addiction to smoking
		Forgetting the female roles	Loss of cooking tastes, regarding less importance for the house cleanliness, regard of less importance for the personal appearance, learning male habits
		Disrupted daily life routines	Disrupted hours and quality of sleeping, loss of food quality, sleeplessness, insomnia, nightmares, fatigue, high burnout
	Social problem	Compulsory isolation	Loss of familial ceremonies, loss of parties, diminishing relations with friends
		Social rejection	Being rebuked by the family, being rebuked by the friends and relatives, being ignored by the family
		Stigmatization	Being stigmatized for having affairs with men, being stigmatized for accompanying male drivers, and for accompanying with the load owner
	Positive Consequences	Financial independence	Acquiring personal income, relative financial affordability, spending of life by insignificant income from Kolberi
		Strengthening scope of social relations	Communication with different people in Iran and Iraq, familiarity with other women, continued communication with Kolbers following doing Kolberi, use of other female Kolbers
		Strengthening identity and social status	Gaining social prestige, self-sufficiency, increasing self-efficacy, increasing self-confidence, higher managerial abilities

## Main category

### ➢ Women's Kolberi: unpleasant coexistence of poverty, environment and patriarchy

Poverty is the most significant factor that can lead to the emergence of Women's Kolberi. A considerable portion of this poverty is related to the economic and social structure of the society, particularly in Kurdish regions, which are among the most deprived areas of Iran. These regions have not experienced significant industrial and occupational growth, and many people suffer from unemployment. As a result, a significant number of people turn to porterage, and women, in particular, have witnessed family members, friends, or relatives engaging in this occupation. Therefore, the observation of this situation has laid the groundwork for women to become involved in porterage. The geographical environment, including border areas and mountainous regions, also plays a role in the formation of porterage but is not the determining factor. Kurdish regions are mostly mountainous and lack fertile lands for agriculture, resulting in high poverty rates. Thus, porterage becomes the only source of income for many people in these areas. Additionally, living in mountainous regions imposes hardships on women, making it easier for them to endure the suffering of porterage. Furthermore, the existence of patriarchal structures in society prevents women from attaining a suitable economic and social status. Many women, especially until a few decades ago, were deprived of educational opportunities in the region due to limited facilities and the prevalence of patriarchal beliefs that confined women to the household and child-rearing responsibilities. Consequently, they lacked vocational skills and became more vulnerable, thus resorting to porterage. Patriarchal structures not only influence women's involvement in porterage but also affect the way society deals with and reacts to porterage. Due to the challenging nature of the occupation, porterage is perceived as a masculine job. Therefore, women attempt to adopt masculine behavior and mannerisms during porterage to minimize judgment and harassment. These three phenomena, namely poverty, environment, and patriarchy, contribute to an unpleasant and undesirable coexistence that gives rise to a phenomenon known as women’s Kolberi, distinguishing it from male porterage.

## Causal conditions

Causal conditions behind the formation of Iranian Kurdish women Kolberi is made up of two components: personal characteristics and economic factors. In fact, part of these conditions pertains to the character and abilities of women, and the other part to the economic conditions, making them have no choice but to turn to Kolberi.

### Individual characteristics

Part of this component pertains to illiteracy and a lack of skills to undertake another occupation, while another part pertains to the individual’s personality characteristics, such as being emotional and gaining independence, which leads women to Kolberi. However, the previous individual’s experience in mountainous areas makes Kolberi's work simpler for them. Also, considering Kolberi ethical is effective.

#### Low empowerment

Most women under study were either illiterate or had no literacy, causing them not to be able to get another job. Also, due to illiteracy and other restrictions, they could not go to other places or gain other skills. For this, they had no choice but Kolberi.

"I am illiterate and cannot do anything else except for Kolberi" (Participant 6, 45 years old, illiterate).

"I know no skills. Other women weave carpets, but I can’t do that" (Participant 11, 53 years old, illiterate).

"Thank God, Kolberi is there to do it." Otherwise, I didn’t do anything else. What should I do?" (Participant 7, 51 years old, illiterate)

#### Personality characters

Some of the female characteristics under study cause them to turn to Kolberi. Since Kolberi is associated with emotion and high risks, only women who have an emotional and risk-taking character can turn to it. Also, since the money from Kolberi can help women gain financial independence, those who are more willing to gain independence from the family and can earn more money in a shorter time are more attracted to this job. Also, due to the excessive hardships of Kolberi, the women who are more diligent and hardworking and can endure the miseries of the routes can turn to it.

"Before doing Kolberi, I used to cook and sell bread. Its money was so low. However, Kolberi is harder, but its money is greater, so I chose it. (Participant 4, 37 years old, diploma).

"From childhood, I had an emotional and adventuristic character. I used to do dangerous work. Kolberi is like this, and since all the to-and-fro routes are full of risks and emotions, I chose it. That’s enjoyable" (Participant 18, 16 years old, under diploma).

"When my husband died, I did not want to make myself and my children dependent on others. For this, I decided to do Kolberi. Generally, I don’t want to take money from anyone. When my husband was alive, I used to do other work, and I was independent" (Participant 20, 44 years old, under diploma).

"It is true that Kolberi is difficult and harms men, but since I used to do hard work from childhood, I am not so harassed by this job. In general, I am a diligent person and never get tired of working" (Participant 19, 39 years old, under diploma).

#### Previous experience

Most women under study were farmers or animal keepers in the past and used to live in mountainous villages where they could commute to the mountainous regions and carry loads there. Also, some of them used to bring water from mountain springs, and they had to travel through lengthy routes in the mountains to get it back by carrying large gallons of water. Thus, most of them had a history of carrying loads from mountainous regions. This was also a factor that led to Kolberi.

"Prior to this, I was living in mountainous villages and spent most of my life there. Kolberi is not hard for me. Sometimes, we used to go to the mountains to pick weed and sell it. Sometimes, we used to carry loads of 50 kilos on our shoulders and bring them down the mountains" (Participant 14, 19 years old, diploma).

When I was a child, I used to get the sheep to the mountains for grazing. There were times when a sheep gave birth or fell from the cliff and broke its legs. I used to piggyback it down from the mountain" (Participant 16, 35 years old, diploma).

"We were trained as mountaineers in childhood. Our villages were one hour from the springs. For this, we had to go through the routes several times, carrying water. All of this made us used to the act of Kolberi. One cannot do the act of Kolberi if s/he has not lived in mountainous regions." (Participant 2, 29 years old, higher than diploma).

"Our village was mountainous, and since cars couldn’t commute there most of the time, we had to carry our own products to get them home. That was not so different from the act of Kolberi; it was not illegal, though, but Kolberi is illegal, as the hardship of both works is the same." (Participant 13, 37 years old, illiterate).

#### Considering Kolberi as ethical

Most Participants, despite the illegality of the act of Kolberi by the Iranian government, stated that Kolberi was not illegal and its money was lawful because most of the women brought goods that can be used in the lives of the people and are basic needs of the people. Also, since Kolberi made the price of goods cheaper, it was a great help to people. They maintained that they were doing a useful job and that it was in the interests of the people of the two countries of Iran and Iraq.

"For me, Kolberi is not a crime because we bring goods that have no harm." We are not carrying drugs or alcoholic beverages." (Participant 15, 42 years old, illiterate)

"Kolberi money is lawful because we endure much hardship for it" (Participant 14, 19 years old, diploma).

"As Kolbers, we help consumers in Iran and Iraq purchase goods at lower prices. For me. Kolberi is useful for both countries, and the people of the villages and cities there confirm this. You can ask people there; they will certainly confirm my words" (Participant 6, 42 years old, illiterate).

"I only get goods I produce myself from Iraq. How on earth can this be a crime? Anyone likes to produce something and sell it at a cheaper price. I am doing the same." (Participant 22, 55 years old, illiterate)

#### Considering Kolberi as a sport or hobby

Because women in Kurdish areas are highly forbidden from spending their free time, and almost in most cities and villages there are no suitable places for leisure, recreation, or sports, some women said that they preferred Kolberi because it is a kind of recreation that makes them active. Also, since the mountains of Kurdistan are beautiful in the autumn and spring, some participants stated that they enjoyed this beauty.

"As Kolberi makes me distance myself from the home environment and go around the mountains, that’s a hobby" (Participant 6, 45 years old, illiterate).

"Kolberi is, in itself, a kind of maintenance spirit." Yes, it is very difficult, but when you do it, you seem to perform some sports exercise" (Participant 24, 33 years old, higher than diploma).

"When I go to do Kolberi in the springs, I can really enjoy the nature. I am not harassed at all. I think I could not go to Kolberi if it were not for the green mountains there" (Participant 8, 17 years old, under diploma).

### Economic  factors

This category is made up of financial poverty and unemployment. The presence of widespread poverty in families, mostly arising from unemployment in Kurdish regions of Iran, causes women to have no choice but to turn to piggybacking.

#### Financial poverty

Most women participating in the research said that poverty and a lack of sufficient income led them to turn to Kolberi. Most of them were from poor families who, without Kolberi, could not meet their ends. Also, low support from social bodies was influential.

"We have neither land nor house. We have rented a house and cannot afford to meet our needs. We need to do Kolberi" (Participant 23, 42 years old, illiterate).

"Life incurs many costs, and we have no income. My children are small and can’t work. I have to go Kolberi" (Participant 17, 38 years old, diploma).

#### Unemployment

Kurdish regions are among the most deprivileged regions of Iran. Due to a lack of adequate investment, there are few industrial workshops in this region. This makes it hard to find a good job. Since these regions are mountainous areas, there are few fertile lands, and people cannot earn a living through farming, thus being forced to Kolberi. In fact, there is a kind of widespread unemployment that causes people to piggyback, with many of the participants stating that they could not turn to Kolberi if there were other alternative jobs.

"There is no other job to do; otherwise, I would never do Kolberi." (Participant 2, 29 years old, higher than diploma)

"If there is someone to come forward and give me a good job, I will do away with Kolberi" (Participant 10, 26 years old, diploma).

"There are no other jobs in here—no factories, no workshops, nothing. Our farming also has little income, and we have to go to Kolberi" (Participant 12, 56 years old, illiterate).

"I have no money to get a job around here." I have to come and do the Kolberi" (Participant 16, 35 years old, diploma).

"I attempted a couple of times to relinquish Kolberi. I looked for a job for quite a while, but I failed. With four children, I can’t remain idle. We will die of hunger" (Participant 23, 42 years old, illiterate).

## Predisposing conditions

The conditions predisposing women to Kolberi are made up of social and cultural factors as well as familial factors.

### Social and cultural factors

This category deals with social learning, cultural interactions, and similarities between Iran and Iraq that lay the groundwork for the female Kolberi.

#### Social learning

Due to large-scale unemployment and poverty in Kurdish regions of Iran, Kolberi has become a solution for earning a living in these regions over the past three decades, as it is now a common job in the areas under investigation. Many of the Participants stated that they turned to Kolberi by observing friends, family, and other members of the community, with others quoted as saying that Kolberi was a family job.

"At first, I didn’t know that women were also Kolberi, until one day one of my friends told me she was Kolberi, asking me if I would go with her. I was quite concerned and sad that I couldn’t do it, but she then persuaded me" (Participant 22, 55 years old, illiterate).

"My brother used to do the Kolberi for years; I was a bit familiar with Kolberi, but I never went to do it by myself until two years ago, when I had to go Kolberi following the death of my husband" (Participant 16, 35 years old, diploma).

"Our house is situated in a border village. I could see many men going to Kolberi every day. It was interesting to me to see what Kolberi would like. I wished to go to Kolberi one day and earn a living. One day, I became acquainted with a woman who used to go Kolberi, which affected me" (Participant 19, 39 years old, under diploma).

#### Social interactions and bonds

Iranian Kurdish regions share borders with Iraqi Kurdish regions, sharing some cultural, linguistic, and social similarities. This issue makes it easier to establish exchanges and communications between them. Also, there are many marital relations between the two cultures, with many travelers commuting between the two countries. One should say, however, that many of the Kurdish people of Iraq came to Iran during the large-scale carnage of the Kurds by Saddam Hussein, the then Iraqi dictator, as they were welcomed by people here. Now that they enjoy better economic conditions, Iranian Kurds frequently commute there.

"I go shopping for peas and beans to sell them in Iraq, where I have several good friends who help me sell them" (Participant 23, 42 years old, illiterate).

When I go to Iraq, I don’t feel like I am in another country. They are all Kurds and understand my language. If they were not Kurds, I could not do the Kolberi so easily" (Participant 7, 51 years old, illiterate).

"I am a friend of several Iraqi families. I always take them the stuff they need. If I stay there for some nights, they respect me so much. They say the Iranian Kurds helped us so much in the past and sheltered us" (Participant 18, 16 years old, under diploma).

"Many of our relatives are in Iraq. For this. It is not difficult for us to go there. If there is a problem over the border, they will come and help us." (Participant 24, 33 years old, higher than diploma)

### Familial factors

This category is made up of a lack of support and family compulsion and refers to familial problems that lead women to Kolberi. Of course, many of the problems arise from economic woes.

#### Lack of supporter

Most Kolberi women were from families with weak financial status, with married women having incapable or crippled husbands. Also, divorced and widowed women were also guardians of the house and had to earn a living by themselves. Thus, they had to turn to Kolberi.

"Before my husband died, I never thought of Kolberi, but when he died, we felt numerous pains and much pressure, and that’s why I had to find a job and had no choice but to do Kolberi" (Participant 11, 53 years old, illiterate).

"My husband fell from an apartment and hasn’t worked since then; he had no insurance, either. I had to work for myself and turned to Kolberi" (Participant 6, 45 years old, illiterate).

"My husband is addicted and can’t work. He does not procure the living for us. So, I turned to Kolberi to change the situation." (Participant 28, 55 years old, under diploma)

#### Familial compulsion

Most participating women stated that they were faced with numerous economic problems in the family, arguing they had a dire situation and had to avoid Kolberi. Most of the women stated that they were from populous families, as the presence of a crippled child could make the situation worse.

"I have four little kids, two of whom are crippled. If I don’t work, they’ll die of the hunter" (Participant 17, 38 years old, diploma).

"Most Kolberi women have a poor life; I have seen no rich woman engaged in Kolberi" (Participant 8, 17 years old, under diploma).

## Intervening conditions

Intervening conditions for the tendency of women to Kolberi are made up of one category of Kolberi advantages and features.

### Advantages and characteristics of Kolberi

With all its difficulties and direct situations, Kolberi also includes some advantages that encourage women to do it. These advantages include an appropriate income, working independence, being addicted, and receiving support during Kolberi.

#### Appropriate income

Despite large-scale poverty and unemployment and a lack of high-income jobs, Kolberi promises better job advantages, and for this reason, women turn to Kolberi for its money.

"Before I turned to Kolberi, I used to weave carpets. I couldn’t get the money for some reasons, but I was paid several months later. You know you can get the money from Kolberi immediately." (Participant 3, 38 years old, under diploma)

"Kolberi is very difficult, but just as you get the load off the car and you get the money that will feel great." (Participant 9, 14 years old, under diploma)

"In other jobs, you have to work days and nights to get paid. After all, you don’t get paid much, but in Kolberi, you get paid soon with high incomes." (Participant 15, 42 years old, illiterate)

"Kolberi entails many risks; however, it’s great for you to get paid, and the income is not that bad." (Participant 22, 55 years old, illiterate)

#### Working independence

Despite all the hardships of Kolberi and the fact that people themselves determine how much time a week and when they should do Kolberi, one would say that people can gain much independence and feel independent. For this reason, some women who want to be independent can work as Kolbers and work at home.

"I have many problems and cannot do another job. For this, I chose Kolberi because you can do it whenever you want" (Participant 19, 39 years old, under diploma).

"Kolberi is good because you both work and protect your children. As in my example, I do it two nights a week" (Participant 4, 37 years old, diploma).

"The advantage of Kolberi is that we can get other work done as well." I can both keep the animals and do the farming. I can go to Kolberi every time I wish. Kolberi does not create any limitations for me, as I can do other things and protect my children besides Kolberi" (Participant 1, 40 years old, illiterate).

#### Being addictive

Some of the Participants stated that they were addicted to Kolberi and could not quit it. Yet even some of those who have gotten richer have failed to quit. One would say that the good income of Kolberi and the lowering of stress and pressure in life due to the familiarity with the existing situations make women tempted to continue Kolberi and return to it off and on even after they have quit it.

"Most of the time, I tell myself this is the last time I go to Kolberi; after a couple of days, I say I can’t quit it." (Participant 17, 38 years old, diploma).

"We Kolbers discuss that this job is like smoking; whoever does it once can’t quit it." (Participant 3, 38 years old, under diploma)

"Kolberi is very difficult; however, if you go to Kolberi once, you can’t quit. It is addictive. You know Kolbers turn to this job for its money." (Participant 11, 53 years old, illiterate)

#### Receive support during Kolberi

Due to their gender and physical situation, women Kolbers are more supported by border guards, load owners, and other Kolbers. When a problem arises for them, they come to help. They are also supported by the buyers of the loads over the borders in Iraq, as their loads are sold sooner.

"Border guards of the two countries also care for us; sometimes they don’t say anything to us, but they get the men Kolbers and confiscate the load from them." (Participant 10, 26 years old, diploma)

"If border guards see us, they just issue warnings because we are women and leave us alone" (Participant 12, 56 years old, illiterate).

"Most load owners take care of us; sometimes they give us lighter loads and pay us more wages. When in Iraq, we are more welcomed by Iraqis because we are women, and that’s why our loads are sold soon" (Participant 18, 16 years old, under diploma).

## Strategies

Strategies used in connection with Kolberi by women fall into the category of strengthening compatibility with Kolberi.

### Strengthening compatibility with Kolberi

This category refers to solutions and interactions that women use to be more compatible with the Kolberi conditions. In fact, women use such solutions as taking male identities, concealment, the formation of female groups, and more communication with God to adapt to the situation.

#### Taking male identity

Since Kolberi has difficult conditions, it is considered a male job. For the Kolberi women to adapt to the situation, they take on male identities, wear male clothes, use male expressions, and smoke, which is a more or less male act.

"Kolberi is a male activity, but we have to do it, too." Sometimes, I think we need to work like men" (Participant 12, 56 years old, illiterate).

"Most Kolbers smoke. When we see males smoking, we do it, too" (Participant 19, 39 years old, under diploma).

"To show we are the same as men, we smoke and talk like men" (Participant 22, 55 years old, illiterate).

"Sometimes, when we cover our faces and wear male clothes and speak like men, others think we are men" (Participant 20, 44 years old, under diploma).

#### Concealment

Since Kolberi is thought of as a male activity in Kurdish regions and fewer women engage in it, and if they turn to it, they are rebuked by others, many of the woman Kolbers try to hide their Kolberi job from the others and even from their own families".

"Sometimes, when I don’t want others to know who I am, I wear male clothes" (Participant 1, 42 years old, illiterate).

"When first going to Kolberi, I used to live alone, far from my family, to get the job hidden" (Participant 5, 52 years old, illiterate).

"For two years, my children didn’t know that I was Kolber; I didn’t want them to know I was Kolber, for I knew they would disagree with it." (Participant 23, 42 years old, illiterate)

"Only families know that I do Kolberi, as my relatives are unaware of this, because if they get to know it, they curse me." (Participant 24, 33 years old, higher than diploma)

#### Formation of female groups

Most of the time, women Kolbers form women's groups to work easily and to be less rebuked by relatives and friends. In this connection, using the counterpart groups, women piggyback along with other groups and try to communicate with each other as far as possible.

"I only go Kolberi with women; I have not associated with women when carrying goods" (Participant 15, 42 years old, illiterate).

"When taking goods over the border to Iraq, most of us try to transact with women, thus avoiding being stigmatised by others." (Participant 17, 38 years old, diploma).

#### More communication with God

Most of the women participating in the research stated that they needed more communication with God to cope with all the stress and fear of Kolberi, praying with their God, and giving parts of their income as alms. This way, they can feel more comfortable.

"I always trust in God because I know He cares for me" (Participant 1, 42 years old, illiterate).

"At nights that I have more fears, I try to protect myself by recourse to God (Participant 3, 38 years old, under diploma)

"Most of the time, when the situation gets worse, I console myself by praying to God" (Participant 5, 52 years old, illiterate).

## Consequences

Women Kolberi entails some consequences due to the social and cultural context of the regions under study; most of these consequences are negative and can endanger the individual and social health of women. In some cases, however, this phenomenon may be associated with positive consequences because having an income and gaining financial independence may make women feel pleased.

### Individual problems

Kolberi in Iran is illegal, and women Kolbers face many fears and hardships in this way, which may compromise their individual health.

#### Physical harms

Women Kolbers have to carry heavy loads when doing this job, as mountainous routes are also dangerous and they may fall from cliffs. Therefore, the carriage of heavy loads causes some bodily problems, including backaches, headaches, knee aches, shoulder dislocations, and even amputations.

"After some time, I felt some pains in my back. I went to a doctor who told me not to do the work" (Participant 7, 51 years old, illiterate).

"I don’t know why, but since I began Kolberi, I have had my headaches more severe, as, at times, I am so annoyed that I wish to die." (Participant 10, 26 years old, diploma)

"I have climbed these mountains with heavy loads so that I am now suffering from knee aches" (Participant 4, 37 years old, diploma).

"One time that I had a heavy load and had fastened it to my back, I slipped and fell with the load. Both my shoulders were dislocated. I couldn’t do the Kolberi again. Now that I am fine, it hurts me. I am uncomfortable with the pains." (Participant 6, 45 years old, illiterate)

#### Mental issues

Since Kolberi is a crime, the female Kolbers suffer from much stress and anxiety, as some border guards do not treat them well and mock them, thus causing them to have low morale. Women Kolbers, fearing being caught and losing their lives, constantly live under stress and worry. In addition, they are also afraid of the perils of the routes and the predatory animals on their way. Since Kolberi is a male activity and women Kolbers hide their job from their relatives, the fact that they get to know that women are doing this job could lead to more humiliation. Also, concerns over what may happen on the way could cause women to lose face, and that’s a permanent concern for women.

"Kolberi makes man crazy because it piles pressure, stress, and anxiety on him" (Participant 23, 42 years old, illiterate).

"Sometimes, border guards treat us very badly and humiliate us. I tell myself I would never go back to Kolberi" (Participant 11, 53 years old, illiterate).

"I am a woman. If someone disrespects me along the way, I will lose face. All my concerns come from the disrespect of others, which may cause me to lose face." (Participant 17, 38 years old, diploma)

"Since I conceal Kolberi from my family, my biggest fear is that they may get to know it. I don’t know how they will treat me, but I am sure they will get angry at me." (Participant 5, 52 years old, illiterate)

"In these mountain ranges where we piggyback, there are animals like wolves, hyenas, and jackals who may hurt us" (Participant 12, 56 years old, illiterate).

#### Loss of life security

Kolber women suffer from many dangers all the way back and forth, which can cause their deaths. On the one hand, the impassable mountain path, compounded with the snow and ice that cover these mountainous areas in winter, and on the other hand, injuries and deaths inflicted by border guards and incidents during carrying goods to the border annually cause high rates of fatalities.

"The mountainous ranges in here are very dangerous. Sometimes we go through paths that, if we are not careful for a moment, we could fall down and die (Participant 8, 17 years old, under diploma).

"Kolberi in the winter is very terrible because there is so much snow and cold. Last year, we were caught in an avalanche, and we were about to die, but we were lucky and survived; however, every year a large number of Kolbers get stuck in the snow and die." (Participant 9, 14 years old, under diploma)

"Sometimes border guards shoot at us, and we might get hit and die" (Participant 18, 16 years old, under diploma).

"Two years ago, I lost my way in the winter at night, and I had to stay in the mountains at night. It was very cold. I was about to die. I was so cold that I couldn’t move my body. After that night, one of my toes got numb and I haven't moved since then (Participant 1, 42 years old, illiterate).

"Usually, when we go to the borders, as many as 10 people get into a van, and we move then, but sometimes there are even more occupants. We had an accident once or twice" (Participant 6, 45 years old, illiterate).

"Two months ago, when I was going to the border for Kolberi with my friend, we had an accident, and I broke my hand. The car had no insurance, and I received nothing." (Participant 19, 39 years old, under diploma)

#### Addiction

Carrying and transporting heavy loads is a very difficult and tedious task, especially for women who are physically weaker than men. Also, since Kolbers have to remain awake for some nights, they need drugs to remain awake, and this results in their addiction.

"To easily carry the goods, I need to take pills" (Participant 18, 16 years old, under diploma).

"To avoid sleeping at night and falling from the cliffs, I use methamphetamine." (Participant 3, 38 years old, under diploma)

"I started smoking when I came for Kolberi; I don’t know if I use drugs to relieve my stress, but Kolbers need to use drugs." (Participant 3, 38 years old, under diploma)

#### Forgetting the female roles

Kolberi is regarded as a male activity due to its hardships. Hence, when women turn to it, harsh working conditions cause them to distance themselves from their female roles and to appear in a male role. As many of the participants stated, they no longer demonstrate female characters.

"Kolberi causes us as women to regard home affairs as insignificant; prior to Kolberi, I used to clean the house and sweep it, but now I can’t do that." (Participant 11, 53 years old, illiterate)

"Previously I used to go for a haircut, but now it’s no longer important how I look" (Participant 12, 56 years old, illiterate).

"I have become like men as we have associated with them. Sometimes, my girl tells me I behave and speak like men." (Participant 28, 55 years old, under diploma)

#### Disrupted daily life routines

Since Kolberi has no definite time limits and is mostly performed at night, it causes the women Kolbers to change their lifestyles. Many of whom suffer from lack of sleep as their diets change over the long run, which may also cause burnout and high working pressure.

"We mostly go for Kolberi at night." For this, we have to remain awake until the morning. Upon returning, we returned at night. Thus, we have to remain awake for several nights. (Participant 3, 38 years old, under diploma)

"We have a lot of stress most of the time. Most of the time when we are at home and sleep, we have nightmares" (Participant 21, 15 years old, under diploma)

"I never managed to comfortably sleep since I chose Kolberi" (Participant 24, 33 years old, higher than diploma).

"We have to eat light food for Kolberi, or sometimes we can’t eat anything" (Participant 18, 16 years old, under diploma).

"Kolberi is really difficult. I always say a woman can never continue as a Kolber, as it is devastating." (Participant 10, 26 years old, diploma)

### Social problems

Due to the social and cultural contexts and gender debates in the regions under study, as well as the harsh Kolberi conditions, women Kolbers are thought to suffer from many social issues, which include compulsory isolation, social rejection, and stigmatisation.

#### Compulsory isolation

Kolberi causes women to have no time to go to special familial ceremonies and parties. For this reason, they are secluded from others.

"Since most of the time I am in the mountains, I can't go to the burial ceremony of my friends; that's why they complain about that later." (Participant 2, 29 years old, higher than diploma)

I have to quit many parties because most of the time my job has no definite time limits" (Participant 9, 14 years old, under diploma)

"Previously, I used to go to parties and visit my friends and relatives more, but now that I'm doing Kolberi, I go fewer times because, on the one hand, I don't have the time, and on the other hand, I'm really tired" (Participant 18, 16 years old, Under diploma).

"I don't go to a lot of family events since I began Kolberi; that's why most of my relatives and family members are unhappy with me" (Participant 7, 51 years old, illiterate)

#### Social rejection

As mentioned above, Kolberi is viewed as a harsh and tedious job, so women who turn to Kolberi are rebuked and humiliated by their family and friends, sometimes being rejected and secluded from the public.

"When my family learned about my Kolberi, they got very angry, thus beginning to rebuke me, but I continued my work anyway." (Participant 12, 56 years old, illiterate)

"When my brother learned about my Kolberi, he came and said, I will give you as much money as you want; just don't go Kolberi anymore, but I didn't agree; since then, he has never talked to me at all." (Participant 7, 51 years old, illiterate)

#### Stigmatisation

Since most of the Kolbers are men and the drivers and buyers are often men, the women Kolbers are stigmatised and humiliated. Sometimes women Kolbers are accused by their male counterparts or other women of having relationships with other men, which may have very bad consequences for them due to the traditional social relations of the region.

"Sometimes I hear bad words from the neighbours, accusing me of having affairs with men" (Participant 16, 35 years old, diploma).

"When we have to get in a car with men and go to the border, some of the relatives have bad feelings about us and slander us" (Participant 17, 38 years old, diploma).

"We have to communicate with men to move and sell the goods we carry, etc., which is why sometimes others misjudge the situation and accuse us of being intimate with men." (Participant 23, 42 years old, illiterate)

### Positive consequences

Despite all its negative consequences for women, Kolberi entails some positive consequences for them, which include financial independence, strengthening of social relations and identity, and improvement of social place. Kolberi may also improve the individual and social lives of women.

#### Financial independence

Women Kolbers earn a relatively good income, which they cannot earn at other jobs. This helps them achieve some kind of financial independence, helping them afford their lives and cope with other problems.

"Kolberi has a good income. It’s really hard, but I am pleased with it because I am no longer dependent on others." (Participant 9, 14 years old, under diploma)

"I earn a living by Kolberi, and it is not required to get money from others" (Participant, 14, 19 years old, diploma).

"Previously, when I used to bake bread, I always had a monthly deficit, and I had to turn to my brothers for money. Now that I am Kolberi, I no longer need it." (Participant 11, 53 years old, illiterate)

#### Strengthening scope of social relations

Women Kolbers need to communicate with many people to exchange and trade their goods, as these communications last for a longer period of time. Also, communication with other Kolbers can help develop their social ties.

"We become friends when we go Kolberi with other women; this friendship is really valuable. We help each other most of the time." (Participant 7, 51 years old, illiterate)

"I have found many friends both in Iran and in Iraq. Most of the load buyers are in Iraq. For this, I communicate with them, and when I can’t return home back in Iran, I can stay at their homes without any fear" (Participant 14, 19 years old, diploma).

"Kolberi helps me find more friends and communicate with a large number of people; this gives me a good feeling." (Participant 2, 29 years old, higher than diploma)

#### Strengthening identity and social status

Besides gaining a good income, Kolberi women's social relations have improved. This increases social prestige as well as the feeling of self-sufficiency, culminating in greater self-efficacy. Also, since some of these women handle loads and other Kolbers, their management abilities are strengthened.

"No one knew me before I went to Kolberi; however, many people now know me and count on me." (Participant 2, 29 years old, higher than diploma)

"I have a good feeling when I see I can cope with life and pay for my expenses without needing anyone." (Participant 9, 14 years old, under diploma)

"I have gained more self-confidence since I began Kolberi. I'm not afraid of anything anymore because I know I can handle everything." (Participant 13, 37 years old, illiterate)

"Besides going Kolberi by myself, several other Kolbers work for me at the same time, and I also have a couple of mules. I feel good when I handle all this." (Participant 28, 55 years old, under diploma)

## Discussion

This research aimed to identify the causes and consequences of Kolberi among Kurdish women in Iran using a grounded theory approach. The findings revealed that Kurdish women turn to Kolberi for a number of reasons, as they consider special strategies to adapt to the Kolberi conditions and experience various negative and positive consequences.

Individual characteristics are one of the causal conditions that help form Kolberi but have not been investigated in previous studies. This reason may come from the effects of gender, because all previous research on Kolberi had been undertaken on men [8, 11]. Part of the individual characters had low female abilities. Since patriarchal views still prevail in Kurdish regions of Iran and an increasing number of girls are prohibited from education each year [20], they may turn to Kolberi to earn a living and be financially independent. Of course, the key reason for women's illiteracy pertains to the deprivation and underdevelopment of these regions, because in parts of Kurdish regions, there are either no girl educational centres or their number is low, with many girls being deprived of education [3]. Although there were many people with high education levels among Kolbers, they had been forced to turn to Kolberi due to a lack of occupational opportunities in the region. Personality characteristics also contribute to the formation of Kolberi, as this research nicely refers to. It is stated that Kolberi is not alone affected by social and economic situations; rather, it is also inspired by some personality characters. Considering the perilous and hard conditions of Kolberi, which represent an example of a risky job, few people have the capacity to tolerate this. Hence, the women who turn to Kolberi are characterized by district personalities, such as being emotional, adventuristic, and risk-taking. Previous contributions have also suggested that personality traits lead to high-risk behaviors [21–23].

Another finding of the research relates to having prior experience. Mountainous conditions in Kurdish regions and living in mountains could help women prepare for a difficult and tedious life under harsh conditions. If these women lived in other parts of Iran, they would not be physically prepared to cope with Kolberi. However, life in the mountains had provided Kurdish women with similar experiences to undertake Kolberi (e.g., bringing water from the river or carrying farm products from the mountains).

Considering Kolberi as ethical was yet another finding of the research, being in line with the findings of Mahmoodi and Moradi (2021), suggesting that Kolberi is not only not an immoral act but also a decent job that helps women earn a living [8]. In research by Moradi et al. (2022), Kolberi was conceived of as a respectable job, and Kolbers preferred it over migration and doing other work [10]. While Kolberi is thought of as an illegal job in Iran, women did not have such a view, viewing it as totally ethical and a necessity to make a living. They believed that Kolberi was much better than prostitution or robbery. They also regarded Kolberi as a useful good because it helped people in Iran and Iraq buy cheap products. Also, such a view originated from the type of goods they used to exchange, because most of the women used to only exchange edibles, never turning to drugs or cigarettes.

Considering Kolberi as a hobby and as a recreation was also another finding of the research. Due to social and cultural deprivations and a lack of training and recreational facilities, women turn to Kolberi to escape the monotonous environments of the house and to have some physical activities. In most studies done in Kurdish regions of Iran, Kurdish women are reported to face many social restrictions [24]. Part of these restrictions pertains to training and recreational facilities in the studied area. In a study by Irandoost et al. (2021), lack of appropriate sports facilities in Kurdish regions is a cause of obesity among Kurdish women [25]. It is noteworthy that turning to Kolberi is due to a lack of facilities and the use of this risky job as a hobby or entertainment, which may cause harm to women.

Economic factors also cause widespread unemployment and poverty, which lead women to do Kolberi. This is the main reason for turning to Kolberi [5, 8, 9]. In the study by Janparvar et al. (2021), unemployment and a lack of farmlands are the major reasons for the formation of Kolberi [2]. Iranian Kurdish regions are among the most deprived areas of Iran and suffer from large-scale unemployment and poverty. In most of these regions, there are no industrial and manufacturing facilities; in the meantime, the lack of fertile lands makes it difficult for people to earn a living. The Iran-Iraq war converted most of the Kurdish lands into minefields, as failure to demine the lands deprived people of engagement in farming. Thus, one of the ways was to turn to Kolberi.

Social learning was one of the predisposing conditions that led women to turn to Kolberi. In the research by Yoosefi Lebni et al. (2020) on child marriage in the Kurdish regions of Iran, it was found that the high number of child marriages in these regions caused this behavior to be reproduced by other women and men [20]. Man is a social being who learns from observation how to behave in society. In view of the fact that Kolberi has turned into a lifestyle in Kurdish areas in recent decades, especially in the border areas, in many families, one person is at least engaged in Kolberi, as other members and relatives also learn this from others and get involved in it in the future to earn a living.

Social interactions and bonds are among the other predisposing conditions behind Kolberi, as this finding was consistent with the research by Mahmoodi and Moradi, 2021 [8]. Similar results were obtained in AleAhmad's study in 2023 [14]. People on both sides of the border in Iran and Iraq are Kurds and have many linguistic and cultural similarities. This increases their interrelationships and makes it easier for both groups to perform their commercial exchange. The subject greatly contributes to the expansion and continuation of Kolberi because the ability to perform the exchanges makes load owners more willing to send loads and continue to use Kolbers.

Another predisposing condition behind women's Kolberi is family factors, including lacking a supporter and family compulsion. In fact, one would say that most Kolber women turn to Kolberi out of compulsion because they are not financially supported by their families, social organizations, or government institutions. Also, in the meantime, in traditional families, women had to play the role of parents for their children and were often living in large families; given all these problems, they inevitably chose Kolberi as the only possible choice. Moreover, widows are barred from remarriage by family members and society due to the prevalent beliefs and norms in the studied society [26]. For this reason, when the husband dies, women take responsibility for taking care of the children alone for the rest of their lives, and since they lack the necessary skills, they have to turn to Kolberi to earn a living and cope with life's problems.

The advantages of Kolberi were recognized as an intervening factor, which was a new finding of this research. This factor should also be added to the findings of previous studies. Although Kolberi is harsh and tedious work, it also entails advantages that make people turn to it. One of these advantages is appropriate income, which indicates Kolberi for women has more income than other jobs, and this is important for women. Working independently is also an advantage for Kolberi. Since women should simultaneously engage in work both inside and outside the home and deal with mother and wife roles, Kolberi may promise an ideal job, as women themselves set the time and hours of carrying the goods, and this is a great advantage. Due to their gender and physical situation, women Kolbers are more supported by border guards, load owners, and other Kolbers. When a problem arises for them, they come to help. They are also supported by the buyers of the loads over the borders in Iraq, as their loads are sold sooner.

Kolberi is viewed as a male job that few women can perform. Thus, women apply a set of strategies in Kolberi that help them strengthen their power of adaptation to it. Taking on male identity was also one of the women's strategies. By mimicking male gestures, women somehow take the place of their male counterparts. Using this strategy, it is determined that they keep themselves free from sexual violence and other harms. Also, the work of Kolberi over a longer period of time causes women to develop male personality traits. Concealment was one of the other strategies that the women used because Kolberi is regarded as an inappropriate job for women due to its conditions. Thus, most of the women conceal their Kolberi from the people around them and even family members, thereby facing much psychological pressure. AleAhmed (2023) also showed that the women in Kolber prefer others not to notice their work [14]. The formation of women's groups was one of the other strategies adopted by Kolberi women. This could protect them from being stigmatized and provide them with safer working conditions because if they do Kolberi with men, they are branded with negative stigmas. The formation of women's groups helps them to work easily and is less rebuked by relatives and friends. More communication with God was also another strategy. Most of the women participating in the research stated that they needed more communication with God to cope with all the stress and fear of Kolberi, praying with their God, and giving parts of their income as alms. This way, they can feel more comfortable [27, 28].

Kolberi has many consequences for women. Personal problems are one of the most notorious consequences. Physical injuries such as back pain and knee pain are among the most important personal problems facing Kolberi women, which is expected as Kolber women climb up and down the mountains frequently. In the study of AleAhmed (2023), physical problems and injuries were among the complications of women [14]. In their research, Zareshahabadi and Mohammadi (2021) examined such problems as back and knee pain, with cases of arthritis also reported [5].

Mental issues were another finding of the research, which were also noted among Kolberi men [11]. However, women are in a more critical condition than men, as they are worried about losing their faces [14]. Also, some of them have worked as Kolbers without having informed their families, thus concealing their activities and incurring much mental pressure. This has also affected their working conditions, leading them to shirk their family responsibilities. Other problems include a lack of individual security, as reported in most studies [5, 11]. Life insecurity includes falling from mountains and facing natural catastrophes, such as avalanches and typhoons; crashes when being sent to the border with non-standard vans; shots fired by the officials; and stepping on land mines. Since Kolbers lack insurance and incur high treatment costs, they give up treatment and are secluded at home.

Addiction was also another finding of the research, which was also a consequence of Kolberi. Considering the hardships of Kolberi and the stress and mental pressure caused by them, some of the women turn to cigarettes and some energetic drugs to get through these difficulties. These issues, however, are mostly noted among men, as other studies have also referred to them [29].

Forgetting about female roles was another finding of the research. The difficulty and harshness of Kolberi caused women Kolbers to move away from their gender-based roles and to liken men’s characters. Moving away from female roles may reduce the likelihood of remarriage among widows and divorced women, though most of the women who live in Kurdish regions cannot remarry following the death of their husbands due to the social and cultural conditions of their regions.

Another consequence of Kolberi facing women is the disruption of their daily routines. Kolber women do this work at night due to the risks they may face, which may affect their sleeping patterns, feeding, and rest. When they are at home, they cannot have a comfortable sleep due to the stress and pressure of work and constantly have nightmares. In terms of food, they have to turn to cold food, which may endanger their health in the future.

Kolberi is not only associated with individual consequences but also results in social problems. One of these problems is compulsory isolation. The hardship of Kolberi and the undetermined time and place of it may force women to fail to have a proper schedule for their lives, thus forcing them to cancel many of the familial ceremonies. However, this is not all of the problems, as they are also faced with other social problems such as social rejection and stigmatization, which may pile pressure on them. This finding is consistent with those of Zareshahabadi and Mohammadi (2021), who suggested that even men Kolbers were under much pressure from their families and relatives. For them, Kolberi is not a human work but rather an animal work [5]. Women’s tendency towards Kolberi, viewed as a male activity, caused them to be rejected by their families and face numerous conflicts. Also, since most people who communicate with women Kolbers are men, this may cause them to be stigmatized as perverts, which, in accordance with the social sensitivities of the society under study, may even cause their deaths. Similar results were obtained in AleAhmad's study in 2023 [14].

Kolberi not only causes negative consequences but also helps create positive consequences. In a situation where the women of the area under study face occupational problems and employment conditions are not available, Kolberi can make them financially independent through its relatively good income. This is more noticeable in married women who are the head of the household, as they are no longer dependent on others because of their income and have the supervision and interference of their relatives reduced in family life.

The strengthening of the scope of social ties is another positive outcome of Kolber. Although Kolberi causes some social limitations for the women, when the women Kolbers inevitably communicate with various people, this may expand their social relations. The strengthening of identity and social place is also another positive outcome of Kolberi. This finding was not consistent with that of Zareshahabadi and Mohammadi (2021), because they had proposed in their research that Kolberi could humiliate and lower the social status of people [5]. This difference may arise from people's gender. Having financial independence, on the one hand, and expanded social relations and simultaneous management of family affairs and working issues, on the other hand, will help strengthen the social identity of women Kolbers, thus increasing their sense of self-efficacy and self-confidence.

## Limitations and Strengths

Since this study addresses for the first time the causes and consequences of Kolberi in women, it can provide comprehensive and useful data to policymakers and social and economic planners so that they can prevent women from turning to Kolberi and then remove their problems. Another point of strength was that since the researchers were from the areas under study, they could easily communicate with the participants and gain their trust. Also, because one of the researchers was a Kolber, he could better understand the problems of women Kolbers. This research, however, suffered from some limitations. The first and probably the most important of which was the absence of statistics and addresses of the participants. Since Kolberi is illegal and women face their own special cultural limitations, researchers faced limitations in identifying and accessing participants; however, they used local trustees to identify several Kolbers. They used snowball sampling to identify other Kolbers to overcome this limitation. The other limitation was the dissatisfaction of some of the women who participated in the research with the recording of the interviews; however, the researchers assured them that their voices would be kept confidential. The last limitation was finding a suitable place for the conduct of the interview because some of the participants were Kolberi without the awareness of their families. Thus, the interview had to be conducted in such a way that no one else would be informed of it.

## Conclusion

The results revealed that women Kolberi is done under the influence of various personal, family, economic, and social factors. For women, this activity entails unique advantages and characteristics that encourage them to continue it. Kolberi among women can be prevented by adopting such measures as training women to gain job skills, providing them with employment conditions, increasing social, financial, and psychological support for women, especially women without husbands, providing employment opportunities for women, creating border markets and establishing the ground for selling their products, creating sports and training facilities for them, and helping them better use their leisure time.

The present study is among the few studies that have addressed the issue of Kolberi, and especially Kolberi among women. Given that there has not been a dedicated study with a qualitative approach on the causes and consequences of Kolberi among women, some findings of the present study are innovative and are presented in some way for the first and can be a source for future studies and to be challenged by other researchers. Previous studies have only identified negative consequences of Kolberi, but this study shows that this phenomenon can help strengthen the financial independence and identity of women. The study results can serve as a basis for future research, especially educational interventions and quantitative studies (surveys) in the field of Kolberi. Given the spread of the Kolberi phenomenon in the border regions of western Iran and the existence of similar phenomena in other parts of the Iran, the results of the study reveal a valuable set of data on the context, interaction, and consequences of Kolberi among women, some of which can be generalized to the phenomenon of men's Kolberi. These results can be used in future policy making in these areas and lead to the provision of solutions and interventions to prevent women from engaging in Kolberi and mitigate its negative consequences and improve the socio-economic situation of the inhabitants of these regions and reduce the growing trend of these phenomena.

## Data Availability

The datasets used and/or analyzed during the current study are available from the corresponding author on reasonable request..
